# A clinical and molecular characterisation of *CRB1*-associated maculopathy

**DOI:** 10.1038/s41431-017-0082-2

**Published:** 2018-02-01

**Authors:** Kamron N. Khan, Anthony Robson, Omar A. R. Mahroo, Gavin Arno, Chris F. Inglehearn, Monica Armengol, Naushin Waseem, Graham E. Holder, Keren J. Carss, Lucy F. Raymond, Andrew R. Webster, Anthony T. Moore, Martin McKibbin, Maria M. van Genderen, James A. Poulter, Michel Michaelides, UK Inherited Retinal Disease Consortium

**Affiliations:** 1 University College London Institute of Ophthalmology, University College London, London, UK; 2 Inherited Eye Disease Service, Moorfields Eye Hospital, London, UK; 3 Section of Ophthalmology and Neuroscience, Leeds Institute of Biomedical and Clinical Sciences, University of Leeds, Leeds, UK; 4 Department of Ophthalmology, St. James’s University Teaching Hospital, Leeds, UK; 5 Department of Electrophysiology, Moorfields Eye Hospital, London, UK; 6 NIHR BioResource - Rare Diseases, Cambridge University Hospitals NHS Foundation Trust, Cambridge Biomedical Campus, Cambridge, UK; 7 Department of Haematology, NHS Blood and Transplant Centre, University of Cambridge, Cambridge, CB2 0PT UK; 8 Department of Medical Genetics, Cambridge Institute for Medical Research, University of Cambridge, Cambridge, CB2 0XY UK; 9 Ophthalmology Department, University of California San Francisco Medical School, San Francisco, CA USA; 10 Bartiméus Diagnostic Centre for Complex Visual Disorders, Zeist, The Netherlands; 11 Department of Ophthalmology, University Medical Center Utrecht, Utrecht, The Netherlands

## Abstract

To date, over 150 disease-associated variants in *CRB1* have been described, resulting in a range of retinal disease phenotypes including Leber congenital amaurosis and retinitis pigmentosa. Despite this, no genotype–phenotype correlations are currently recognised. We performed a retrospective review of electronic patient records to identify patients with macular dystrophy due to bi-allelic variants in *CRB1*. In total, seven unrelated individuals were identified. The median age at presentation was 21 years, with a median acuity of 0.55 decimalised Snellen units (IQR = 0.43). The follow-up period ranged from 0 to 19 years (median = 2.0 years), with a median final decimalised Snellen acuity of 0.65 (IQR = 0.70). Fundoscopy revealed only a subtly altered foveal reflex, which evolved into a bull’s-eye pattern of outer retinal atrophy. Optical coherence tomography identified structural changes—intraretinal cysts in the early stages of disease, and later outer retinal atrophy. Genetic testing revealed that one rare allele (c.498_506del, p.(Ile167_Gly169del)) was present in all patients, with one patient being homozygous for the variant and six being heterozygous. In trans with this, one variant recurred twice (p.(Cys896Ter)), while the four remaining alleles were each observed once (p.(Pro1381Thr), p.(Ser478ProfsTer24), p.(Cys195Phe) and p.(Arg764Cys)). These findings show that the rare *CRB1* variant, c.498_506del, is strongly associated with localised retinal dysfunction. The clinical findings are much milder than those observed with bi-allelic, loss-of-function variants in *CRB1*, suggesting this in-frame deletion acts as a hypomorphic allele. This is the most prevalent disease-causing *CRB1* variant identified in the non-Asian population to date.

## Introduction

To date, more than 150 disease-associated variants in *CRB1* (OMIM #604210) have been described, associated with a range of inherited retinal disease (IRD) phenotypes including Leber congenital amaurosis (LCA), early as well as adult-onset retinitis pigmentosa (RP)—with and without a Coats-like vasculopathy, and more recently macular dystrophy and foveal schisis [1–11]. Characteristic features of *CRB1*-associated retinopathy include early onset maculopathy, loss of retinal lamination with increased retinal thickness, nummular intraretinal pigmentation, preservation of the para-arteriolar retinal pigment epithelium, and the presence of macular cysts [12]. Expression of the retinal phenotype, however, is variable, even within families, and a number of either genetic or environmental factors have been postulated [13].

*CRB1*, a human homologue of the *Drosophila melanogaster* gene crumbs (*crb*), is expressed in the foetal brain and the inner segments of photoreceptors in humans [2, 14]. It consists of 12 alternatively spliced exons, resulting in two different transcripts of 1376 and 1406 amino acids. Both contain extracellular domains (19 epidermal growth factor (EGF)-like domains, three laminin A globular (AG)-like domains and a signal peptide), but the longer isoform additionally contains transmembrane and cytoplasmic domains (FERM-binding domains and a PDZ-binding motif), which facilitate assembly of adherens junction complexes and linking to the actin cytoskeleton [15, 16]. Consequently, CRB1 has been implicated in mechanisms that control cell adhesion, polarity and intracellular communication, and is considered crucial for photoreceptor morphogenesis and subsequent function [17–19]. In the developing retina, core Crumbs complex proteins localise to the apical side of the epithelium, which will ultimately form the junction between photoreceptor cells and Müller glia, constituting the external limiting membrane (ELM) [14, 20, 21].

To date, no genotype–phenotype correlations have been identified, and a comprehensive understanding of CRB1 function is still sought. This study aims to make advances in this field, presenting novel clinical data describing a specific consequence of the *CRB1* variant c.498_506del.

## Materials and methods

### Subjects and clinical assessment

Patients known to the eye clinic at one of two hospitals (Moorfields Eye Hospital, London and St. James’s University Teaching Hospital, Leeds) with a diagnosis of macular dystrophy and at least one variant in *CRB1* were identified using in-house databases (OpenEyes^TM^, London and Medisoft^TM^, Leeds). Initial searches did not select for bi-allelic variants only to ensure any cases with missing second alleles were not filtered out. Electronic healthcare records and case notes were then reviewed. Patients had been diagnosed by one of the authors on the basis of slit lamp examination and imaging studies including colour fundus photography (Topcon TRC-NW400, Topcon, Japan), spectral domain optical coherence tomography (SD-OCT) and fundus autofluorescence (FAF) (Spectralis HRA and OCT system, Heidelberg Engineering, Heidelberg, Germany). Electroretinography was performed incorporating the International Society of Clinical Electrophysiology of Vision (ISCEV) standards, and included full-field electroretinogram (ERG), pattern electroretinogram (PERG) and electro-oculogram (EOG) [22, 23].

### Next-generation sequencing

Molecular testing was performed either by targeted next-generation sequencing (National Genetics Reference Laboratory, Manchester, UK or Yorkshire Regional Genetics, Leeds, UK), or as part of a national collaborative whole-genome sequencing project (NIHR BioResource Rare Diseases Study) [24, 25]. Segregation studies were performed, where additional family members were available. All patients had previously provided informed consent as part of a genetics research project approved by the local research ethics committee, and all investigations were conducted in accordance with the principles of the Declaration of Helsinki. All *CRB1* variants reported in this paper have been deposited into the ClinVar database at the National Centre for Biotechnology Information under accession numbers ClinVar:SCV000611552-SCV000611557.

### Identification of additional patient

Our search for additional patients who were homozygous for the in-frame deletion (c.498_506del) identified one further individual from the Diagnostic Centre of Bartiméus, Zeist, the Netherlands. In this patient, OCT was performed with Cirrus high definition (Carl Zeiss Meditec, Inc, Dublin, CA), FAF with Canon CX-1 Digital retinal camera (Canon Inc. Shimomaruku 3-CHOME Ohta-Ku, Tokyo, Japan). Electrophysiological examinations included full-field ERG, EOG and multifocal ERG, all according to ISCEV standards [22, 23]. Mutation analysis was performed at the Academic Medical Centre, Amsterdam by targeted next-generation sequencing.

## Results

Seven unrelated individuals were identified with a macular dystrophy due to suspected bi-allelic variants in *CRB1*. No patients with single, heterozygous variants in *CRB1* were knowingly excluded. All patients were of European ancestry and from non-consanguineous pedigrees. In six out of the seven cases, only a single affected family member was identified. MEH3 was the exception, as two great paternal uncles had been diagnosed with presumed autosomal recessive, severe, early onset RP, but had not undergone genetic testing.

At presentation, patients were either asymptomatic, and their disease was discovered by their optometrist (Leeds 1, MEH3 and BDC6), or they were aware of a change in the quality of their central vision, and actively sought medical attention. No patients reported symptoms that were consistent with night blindness or peripheral field loss. Patients presented with bilateral disease in all cases; however, the functional consequences were occasionally asymmetrical (Table 1). The median age at presentation was 21 years (IQR = 19), associated with a median visual acuity of 0.55 decimalised Snellen units (IQR = 0.43). Six of the seven patients were reviewed more than once, with follow-up ranging from 1.5 to 19 years. By the final visit, the median acuity was 0.65 decimalised Snellen units (IQR = 0.70). Over the follow-up period, only two eyes (two patients, MEH2 and 5) recorded a minimal loss of acuity. Across the group, however, older patients tended to have poorer visual function than younger ones (Table 1).

**Table 1:** Genotype–phenotype correlations for *CRB1*-associated maculopathy cases in this study

Case (age at presentation) (gender)	Follow-up (years)	Visual acuity (decimalised Snellen-RE, LE)		ERG	OCT		FAF	Allele 1	Allele 2
		Presentation	Final	Abnormal PERG, normal FFERG	Retina	Δ Subfoveal choroidal thickness (years)			
Leeds 1 (21) (F)	19	0.5, 0.6	0.5, 0.5	Yes	Widespread loss of outer retinal structures throughout the macula with relative sparing of the foveola.	−7 μm RE, −7 μm LE (2)	Central macular hypoAF surrounded by a diffuse region of hyper AF which extends nasal to the disc	c.498_506del, p.(Ile167_Gly169del)	c.4141C>A, p.(Pro1381Thr)
MEH1 – gc20630 (8) (M)	3	0.8, 0.66	1, 0.8	Yes	EZ and ELM seem okay, cysts start in the axonal layer of ONL = Henle layer, temporal loss of volume. Resolve over time and VA improves.	+10 μm RE, +12 μm LE (3)	Central macular hypoAF surrounded by a diffuse region of hyper AF and ill-defined hyper AF ring	c.498_506del, p.(Ile167_Gly169del)	c.2688T>A, p.(Cys896Ter)
MEH2 – gc17649 (12) (M)	9	1.25, 0.25	1, 1	Yes	Perhaps earlier than expected loss of ELM (see LE 2010).	+3 μm RE, +15 μm LE (5)	Limited, hyper AF (similar to MacTel)	c.498_506del, p.(Ile167_Gly169del)	c.2688T>A, p.(Cys896Ter)
MEH3 – gc17311 (30) (M)	2	0.25, 0.5	0.25, 0.5	Yes	ONL collapse (perifovea>fovea), ELM loss in atrophic regions with relative preservation in adjacent less affected zones, no cysts at any of these later stages	+12 μm RE, −4 μm LE (2)	Central macular hypoAF surrounded by a diffuse region of hyper AF which extends nasal to the disc	c.498_506del, p.(Ile167_Gly169del)	c.1431delG, p.(Ser478ProfsTer24)
MEH4 – gc22882 (24) (F)	0	0.5, 0.1	N/A	Yes	Widespread loss of ONL/ELM/EZ structures with well defined transition zone beyond which these structures are relatively spared	N/A	Reduced central macular AF surrounded by a ring of hyper AF that extends nasal to the disc, itself surrounded by a region of more diffuse hyper AF.	c.498_506del, p.(Ile167_Gly169del)	c.2290C>T, p.(Arg764Cys)
MEH5 (29) (M)	1	0.1, 0.16	0.07, 0.16	Yes	Widespread loss of ONL/ELM/EZ structures, thickening and delamination evident between disc and fovea. Epiretinal membrane LE	−13 μm RE, −11 μm LE (1)	Reduced central macular AF that extends nasal to the disc	c.498_506del, p.(Ile167_Gly169del)	c.584G>T, p.(Cys195Phe)
BDC6(10) (F)	1.5	0.8, 0.6	0.8, 0.8	Normal FFERG and EOG, PERG not done. MfERG central ring decreased responses	Foveolar preservation of EZ and ELM. Intraretinal cysts in INL and ONL	Not done	Subtle hypoAF in fovea surrounded by slight hyper AF	c.498_506de1, p.(Ile167_Gly169del)	c.498_506de1, p.(Ile167_Gly169del)

**Fig. 1:**
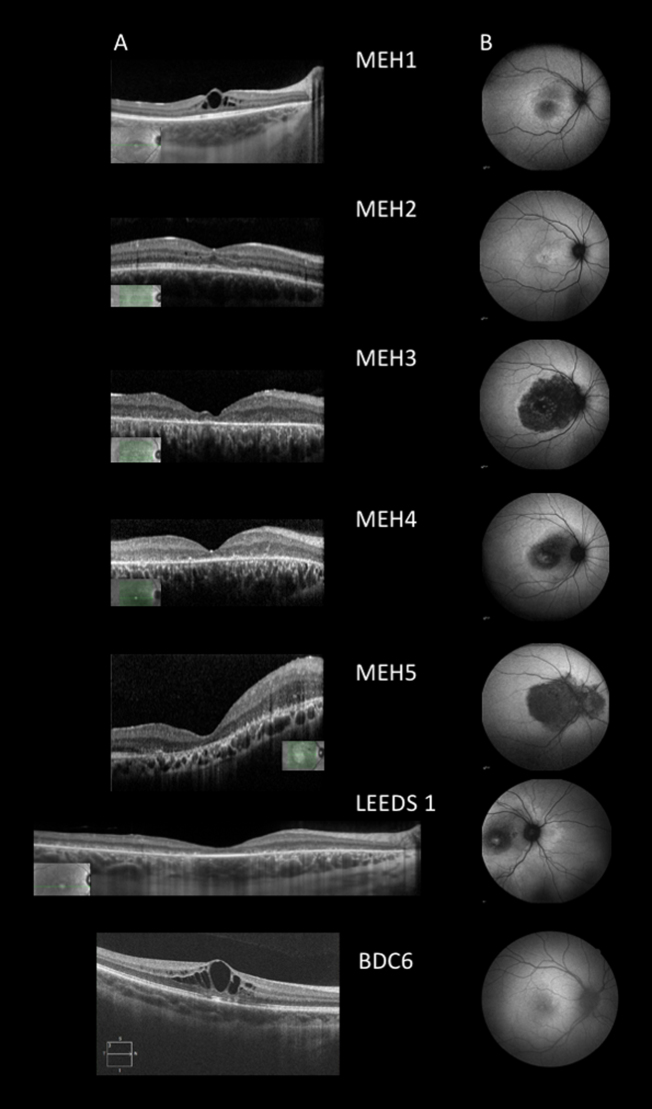
Optical coherence tomography and fundus autofluorescence imaging of patients with *CRB1*-associated maculopathy. **a** Optical coherence tomography (OCT) line scans with near infra-red reflectance images showing scan position (inset). Macro and microcystic oedema is evident in patients MEH1, 2 and BDC6. Foveolar preservation of the ellipsoid zone is present in MEH1, MEH2, Leeds 1 and BDC6. More significant degeneration has already occurred in MEH3 and 4, including loss of the external limiting membrane and outer nuclear layers. Varying degree and extent of macular thickening and loss of lamination is evident in MEH3, 4 and 5. **b** Fundus autofluorescence images from the same time point as OCT scans. Macular autofluorescence is abnormal in all cases, but again with a degree of foveolar sparing. In the oldest patients, the zone of abnormal autofluorecence extends to include the peripapillary retina—initially an increase in signal (Leeds 1), which is likely to evolve to reduced (MEH4) and then lost autofluorescence (MEH3 and 5)

SD-OCT scans identified anatomical changes in all cases (Fig. 1). In the three youngest patients (MEH1, 2 and BDC6), intraretinal cysts were evident in the inner and outer nuclear layers (INL, ONL), a feature that was less apparent with increasing age. In older patients (Leeds 1, MEH3, 4 and 5), there was evidence of outer retinal degeneration and macular atrophy. This initially appeared to spare the foveola, preferentially affecting the perifoveal retina, resulting in a bull’s-eye maculopathy phenotype (Fig. 1). Three patients showed varying degrees of retinal thickening and loss of physiological lamination (MEH3, 4 and 5) (Fig. 1). No patients had evidence of significant progressive choroidal thinning. Macular autofluorescence was abnormal in all patients (Fig. 1 and Table 1). The peripheral retina however retained physiological levels of autofluorescence, although in the oldest patients (Leeds 1, MEH3, 4 and 5) the hyper-autofluorescent signal extended nasal to the optic disc (Fig. 1).

Macular dysfunction was present in all cases, with either low amplitude P50 PERG (*n* = 6) or central mfERG waveforms (*n* = 1). No full-field ERG or EOG abnormalities were observed.

Genetic testing identified two disease-causing variants in all patients. Six shared a rare, single allele resulting in an in-frame deletion of three amino acids (NM_201253.2:c.498_506delAATTGATGG, NP_957705.1:p.(Ile167_Gly169del)) previously associated with disease (rs398124615) [9, 10, 26, 27]. The *trans*-acting variants in each case were predicted to result in either a missense (p.(Cys195Phe), p.(Arg764Cys) or p.(Pro1381Thr)) or a premature termination codon (p.(Cys896Ter), p.(Ser478ProfsTer24)) (Table 1). Patient BDC6 was homozygous for the in-frame deletion (c.498_506del, p.(Ile167_Gly169del)).

## Discussion

The present work describes seven patients with isolated macular disease consequent upon bi-allelic variants in *CRB1*, six of whom are heterozygous for one rare allele (c.498_506del, p.(Ile167_Gly169del)), and one patient who is homozygous. These findings provide the first evidence of a genotype–phenotype correlation in *CRB1*-associated retinopathy, and that in humans the subtlest sign of CRB1 dysfunction is confined to the posterior pole, centred on the macula, that intriguingly initially spares the foveola.

Isolated macular disease is a recently identified and rare consequence of variants in *CRB1* (Supplementary Table 1) [7–10]. The underlying genotypes are summarised in Supplementary Tables 1 and 2. Of all, *CRB1* alleles identified to date that affect protein function, the most prevalently reported in IRD cohorts is p.(Cys948Tyr) (rs62645748)^2^, for which the carrier frequency in the general population is 0.041% (56/276,322 alleles, gnomAD Browser, accessed 1.6.17), and 0.08% in Europeans (50/56 alleles are European). The variant that is the focus of this study, p.(Ile167_Gly169del), is in fact over 1.5 times more common, with a population prevalence of 0.124% (173/277,040 gnomAD Browser, accessed 1.6.17), and again the majority are Europeans alleles (125/173). This variant has never been associated with LCA, and has only been identified in patients with relatively less severe forms of generalised retinal disease (Supplementary Table 2). Its presence has also been used to explain the milder retinopathy evident in one member of a pedigree harbouring three separate *CRB1* alleles—the individual with early onset RP (III:6) was homozygous for p.(Cys948Tyr), while her sister (III:4), who exhibited late-onset disease and a slower progression, carried the in-frame deletion (p.(Ile167_Gly169del)) paired with p.(Cys948Tyr) [27]. The present data, together with that already published, provide a persuasive argument that this in-frame deletion acts as a hypomorphic allele.

Opposing this hypothesis is a prior report of a patient (RP-1426) homozygous for p.(Ile167_Gly169del), but associated with an 'early-onset RP' phenotype [26]. No further clinical details were presented, and it is unknown if this individual harbours additional genetic variants that contribute to the phenotype. Motta et al. also identified a patient harbouring this allele, with an apparently syndromic form of disease (Patient 14—nyctalopia, myopia, glaucoma and hearing loss). As the second, convincingly disease-causing variant remains elusive, it remains uncertain if c.498_506del contributes to this phenotype at all [28]. The gnomAD database (accessed 1 June 17) additionally contains one further individual who is homozygous for this in-frame deletion. As data from individuals with severe, congenital paediatric disorders are excluded from gnomAD, this suggests that here at least this genotype is not associated with LCA. While the degree of overall CRB1 dysfunction is highly likely to influence the ensuing retinopathy, as recently suggested by Motta et al. [28], our data suggest, for the first time, that specific alleles are able to exert a strong influence on the phenotype. In an attempt to clarify the clinical consequences associated with this genotype, this work provides a detailed characterisation of one individual who is homozygous for the c.498_506del variant (BDC6). In this individual at least, this genotype is associated with later-onset (i.e., not infantile-onset), isolated macular disease. When the same variant is paired with a null allele (e.g., in MEH2 and 3), a similarly limited form of disease ensues. As null alleles have never previously been associated with isolated macular disease (Supplementary Table 1), it is likely that this phenotype is determined by the hypomorphic, *trans*-acting variant (Supplementary Table 2). Additional genetic modifying factors may also exist, which could include those in *CRB2*, as rescue of the retinal phenotype in *Crb1* knockout mice has recently been demonstrated using AAV-*Crb2* gene therapy [29]. As CRB1 is only one member of a larger, multimeric protein complex that includes other transmembrane proteins (CRB2, CRB3), which physically interact with a number of cytoplasmic proteins (MPP5/PALS1, PATJ, MUPP-1, MPP3 and MPP4), dysfunction in any one of these could potentially contribute to the disease phenotype [7, 30–33]. The recent discovery that bi-allelic variants in *CRB2* results in syndromic disease, and that retinal dysfunction is identified in a minority of these patients is in keeping with the above hypothesis [34–36].

Other hypomorphic alleles are also likely to exist and Supplementary Table 2 highlights two further examples. Both variants (p.(Gly123Cys) and p.(Arg1331Cys)) introduce a cysteine residue into different EGF-like domains of CRB1. These motifs are characterised by the presence of six highly conserved cysteines, resulting in three pairs of disulphide bridges; introducing a seventh residue is likely to induce structural change, reducing steric flexibility. The specific location where this occurs will determine the overall effect on tertiary structure, the subsequent binding affinity with other CRB-complex proteins and ultimately the disease phenotype. It is also tempting to speculate that further alleles with intermediate pathogenicity also exist—somewhere between those associated with the in-frame deletion and those that are functionally null. In keeping with this hypothesis, when observed in the homozygous state, both p.(Pro836Thr) and p.(Ser740Phe) appear to cause macular dysfunction, but with additional selective impairment of peripheral cones, sparing peripheral rod photoreceptors [8, 13]. It is likely therefore that an allelic hierarchy exists, and that detailed clinical phenotyping will enable this order to be established.

The phenotypes associated with the in-frame deletion p.(Ile167_Gly169) as well as other, 'mild' variants in *CRB1* are also intriguing [6, 7, 9, 10, 26]. First, 'cystic' cavities in the ONL and INL are observed as an early feature of disease. These may be associated with either a qualitative or quantitative reduction in acuity, which appears to fluctuate as the disease evolves (MEH1). It is pertinent to note that intraretinal cysts are also a prominent feature of another disorder strongly associated with Müller cell dysfunction—Macular Telangiectasia (MacTel) [37]. A spontaneously arising rat model for MacTel exists, also shown to harbour a homozygous in-frame deletion in *Crb1*, which results in a limited, macular phenotype [38]. Second, although early macular atrophy is a feature of *CRB1*-associated LCA, the mildest form of disease appears to spare the foveola, presenting as a bull’s-eye maculopathy (Leeds 1, MEH3). Why the disease evolves in this pattern is unknown, but it may relate to structural or metabolic differences between foveolar cones and their parafoveal neighbours. Alternatively, a bull’s-eye pattern of degeneration may be determined by Müller cells, as they too exhibit regional differences in structure. This pattern of degeneration again is observed in the initial stages of MacTel, where the earliest changes of disease are evident temporal to, and not at, the fovea [37]. Third, the extent of retinopathy in the oldest patients appears not to be anatomically limited to the macula, as the retina superior, inferior and nasal to the optic nerve is also affected (Fig. 1). This is an unusual and infrequently encountered pattern of degeneration, but one that is shared with other monogenic retinopathies, where the underlying gene regulates cell–cell adhesion (*ADAM9*, *CDH3*) [39, 40]. Fourth, retinal thickening and loss of lamination may be evident, another phenotypic clue suggestive of *CRB1*-associated retinopathy [41]. Lastly, although not a feature of patients in this series, two independent groups have identified patients with *CRB1* variants and subtle full-field ERG abnormalities, with mild dysfunction evident in cone photoreceptors [8, 10]. If mild dysfunction in CRB1 is associated with localised disease at the macula/posterior pole, then alleles of slightly greater pathogenicity may additionally disrupt peripheral cone, but not rod, function. The most severe variants however result in early and widespread loss of both types of photoreceptors.

In summary, we describe the phenotypic consequences of bi-allelic variants in *CRB1*, where one allele is c.498_506del, p.(Ile167_Gly169del), and the phenotype of one individual homozygous for this variant. This is the most prevalent disease-causing *CRB1* variant identified in the non-Asian population to date. It is more likely to result in localised rather than generalised retinal dysfunction, and so consequently is associated with a better clinical prognosis, as seen in this study. Understanding how genetic variation in *CRB1* contributes to patients’ retinal phenotype will become increasingly important as we continue to develop therapies, and search for biomarkers that will be useful in monitoring response.

## Electronic supplementary material

Supplementary Tables 1 & 2
