# Supplementary material for: A clinical and molecular characterisation of CRB1-associated maculopathy
Source: Eur J Hum Genet. 2018 Feb 1;26(5):687–94. doi: 10.1038/s41431-017-0082-2 (PMC5945653; doi:10.1038/s41431-017-0082-2)
Supplement: Supplementary file 1 — Supplementary Tables 1 & 2 [file 41431_2017_82_MOESM1_ESM.doc]

**A clinical and molecular characterisation of *CRB1*-associated maculopathy – Supplementary Data**

Kamron N Khan, Anthony Robson, Omar AR Mahroo, Gavin Arno, Chris F Inglehearn, Monica Armengol, Naushin Waseem, Graham E Holder, UK Inherited Retinal Disease Consortium, Keren J Carss, Lucy F Raymond, Andrew R Webster, Anthony T Moore, Martin McKibbin, Maria M van Genderen, James A Poulter^*^ and Michel Michaelides^*^.

**Supplementary Table 1. Published cases of bi-allelic variants in *CRB1* associated with a macular dystrophy phenotype.**

| **Published Case** | **Diagnosis (observations)** | **Allele 1** | **Allele 2** |
| --- | --- | --- | --- |
| Tsang *et al.*^7^ | Macular Dystrophy (unusual 5-year progression of macular atrophy). | c.4142C>T, p.(Pro1381Leu) | c.3991C>T, p.(Arg1331Cys) |
| Wolfson *et al.* ^8^ | Maculopathy with cystoid macular oedema. | c.2506C>A, p.(Pro836Thr) | c.2506C>A, p.(Pro836Thr) |
| Shah *et al.* ^9^ | Isolated maculopathy | c.493_501del9, p.(Ile167_Gly169del) | c.584G>T, p.(Cys195Phe) |
| Vincent *et al.* ^10^  Family A  Family B | Familial Foveal Retinoschisis | c.2483G>A, p.(Cys948Tyr)  c.498_506del, p.(Ile167_Gly169del) | c.367G>T, p.(Gly123Cys)  c.2290C>T, p.(Arg764Cys) |
| Sanchez-Alcudia *et al.* ^27^ | “Macular and RPE atrophy” (CF aged 70) | c.498_506del, p.(Ile167_Gly169del) | c.2483G>A, p.(Cys948Tyr) |
| Zhao *et al.* ^38^ | Macular telangiectasia type 2 | c.1685_1698delinsCAAGATGG; p.(Asn562_Gly566delinsThrArgTrp) | c.1685_1698delinsCAAGATGG; p.(Asn562_Gly566delinsThrArgTrp) |

| **Phenotype (1+2)** | **Allele 1**  **[gnomAD frequency]** | **Allele 2**  **[gnomAD frequency]** | **Allele 3 (null/”severe missense”) [gnomAD freq]** | **Phenotype (2+3)** | **Allele 2 hom phenotype** |
| --- | --- | --- | --- | --- | --- |
| Macular dystrophy  (Sup. Table 1) | p.(Ile167_Gly169del) [173/277040] | p.(Ile167_Gly169del) [173/277040] | p.(Cys383Serfs*66) [not seen]  p.(Cys896Ter) [8/277170]  p.(Cys948Tyr) [56/276322] | “EORD”^26^  “EORD” / MD^26^  milder EORD (patient III.6)^27^ | “EORD” (ie. not LCA)^42^  Macular dystrophy (this study) |
|  |  | p.(Ser478ProfsTer24)  [not seen] | - |  | unknown |
|  |  | p.(Pro1381Thr)  [not seen] | p.(Gly850Ser) [6/276848] | LCA^6^ | unknown |
|  |  | p.(Cys896Ter)  [8/277170] | p.(Cys896Ter) [8/277170] | LCA^43^ | LCA^43^ |
|  |  | p.(Arg764Cys) [22/276628] | p.(Ser403Ter) [not seen]  p.(Gly827Ter) [not seen]  p.(Cys948Tyr) [56/276322]  p.(Glu995Ter) [not seen] | LCA^4, 11^ | unknown |
|  |  | p.(Cys195Phe)  [4/277056] |  |  | unknown |
|  | p.(Gly123Cys)  [not seen] | p.(Cys948Tyr)  [56/276322] | p.(Ile205Aspfs*13) [8/245706]  p.(Arg526Ter) [9/277150] | LCA^26^  LCA^105^ | LCA^6^ |
|  | p.(Arg1331Cys) [5/246184] | p.(Pro1381Thr)  [not seen] | p.(Gly850Ser) [6/276848] | LCA^105^ | unknown |
|  | | p.(Pro836Thr)  [not seen] | p.(Cys243Ter) [not seen] | EORP | Foveal schisis plus full field cone abnormalities  Early onset RP ^13^ |
|  |  | p.(Ser740Phe)  [not seen] | - |  | Functional data suggests similar to p.Pro836Thr hom – macular and mild cone abnormalities^8, 13^ |

**Supplementary Table 2: Comparison of phenotypes associated with different combinations of *CRB1* variants.**
